# Biodevelopmental Correlates of Sexual Orientation in Men: Evidence from a Polish Sample

**DOI:** 10.1007/s10508-024-03018-w

**Published:** 2024-11-25

**Authors:** Monika Folkierska-Żukowska, Wojciech Ł. Dragan

**Affiliations:** 1 Department of Psychology, University of Toronto Mississauga, Room 4036, Deerfield Hall, 3359 Mississauga Rd., Mississauga, ON L5L 1C6 Canada; 2 Institute of Psychology, Jagiellonian University, Kraków, Poland

**Keywords:** Male sexual orientation, Handedness, Digit ratio, Familiality, Childhood gender nonconformity, Fraternal birth order

## Abstract

Biological mechanisms proposed to play a role in the development of sexual orientation in men include hormonal, genetic, and immunological factors. The posited roles of these factors are not mutually exclusive; instead, they may be at play to different degrees in different individuals. Direct measurement of these influences is challenging; thus, researchers rely on putative markers. We collected data on five well-established markers in a sample of gay and heterosexual men. We then (1) compared the levels of those markers in gay and straight men, (2) identified latent profiles based on those markers, and (3) compared the proportions of gay and straight men within the profiles. Gay men reported less gender conformity in childhood, a higher proportion of older brothers, were more right-handed, had more non-heterosexual relatives, and had more feminized digit ratios. Of the six identified profiles, the most numerous, containing a significantly higher proportion of straight men, had masculine digit ratios, masculine behavior in childhood, and was the most right-handed. Proportions of gay and straight men did not differ in the profile with the most feminine digit ratio, the profile associated with the highest proportion of older brothers, and the profile associated with left-handedness. Two remaining profiles, associated with familiality, and the most feminine childhood gender behaviors, consisted predominantly of gay men. The study suggests that further investigations of differences within sexual orientation categories are warranted.

## Introduction

Several biological mechanisms, including hormonal, genetic, and immunological factors, have been proposed to play a role in the development of sexual orientation in men (Bogaert & Skorska, 2020). It is important to note that hypotheses relating to these factors are not necessarily competing or mutually exclusive. Instead, growing evidence suggests that gay men are not a homogenous group, and these factors may be at play to different degrees in different individuals (Blanchard & Bogaert, 2004; Folkierska-Żukowska et al., 2020; Swift-Gallant et al., 2019a, b; VanderLaan et al., 2023). Therefore, it is of value to not only study those factors in isolation, but also to investigate how they converge or diverge within and between different individuals of the same sexual orientation to help paint a more comprehensive picture of how they operate. Key questions include whether these mechanisms are additive or constitute separate neurodevelopmental pathways leading to a similar phenotype. Variability in mechanisms may account for inconclusive results in studies that focus on a single mechanism only.

It is challenging to measure these influences directly, so researchers often rely on putative biodevelopmental markers of these mechanisms that are easier to assess. In the present study, we collected data on five such well-established markers in a sample of gay and heterosexual men: handedness, digit ratio, childhood gender nonconformity, fraternal birth order, and familiality. In an attempt to paint a more holistic picture of the relations between them, we (1) performed simple comparisons to see whether gay and heterosexual (straight) men differ on those markers, (2) used latent profile analysis (LPA) to identify latent profiles based on those markers (i.e., separating the participants into groups using a data-driven approach), and (3) performed comparisons of compositions of the identified groups to see whether the proportions of gay and straight men differ within the profiles. The choice of the markers to consider has been dictated by the previous literature described below.

### Handedness

Handedness has been proposed as a potential marker of both prenatal androgen exposure and possible genetic mechanisms (Arning et al., 2015; Ellis et al., 2017; Paracchini & Scerri, 2017; Yeo et al., 1993). It is estimated that men have 20% greater odds of being non-right-handed than women (Papadatou-Pastou et al., 2008). The relationship between sexual orientation and handedness is complex. Non-heterosexuality in men has been linked to both left-handedness and extreme right-handedness (Bogaert et al., 2007; Ellis et al., 2017; Kishida & Rahman, 2015; Lalumière et al., 2000), suggesting a possible curvilinear relationship. Moreover, an interaction has been previously found between handedness and fraternal birth order—i.e., having older brothers increases the odds of same-sex attraction only in right-handed men (Blanchard et al., 2006; Bogaert et al., 2007) or moderately right-handed, but not in men who are non-right- handed or extremely right-handed (Bogaert et al., 2007). Another interesting finding indicates that non-right-handedness is associated with greater gender nonconformity in gay men, but this relationship has the opposite direction in heterosexual men (Swift-Gallant et al., 2017). As non-right-handedness is proposed to be associated with lower prenatal androgen exposure (Ellis et al., 2017), the interaction between sexual orientation and childhood gender nonconformity found in studies regarding handedness could be partially explained by levels of androgenization (Skorska & Bogaert, 2017). Handedness has also been found to be related to the genes encoding the androgen receptor present on the X-chromosome (Arning et al., 2015).

### Digit Ratio

Androgens (including testosterone) are responsible for many sex and gender-related differences, with higher levels of testosterone being associated with more male-like characteristics (Arnold & Breedlove, 1985). The androgen influence hypothesis posits that gay men are exposed to lower-than-average prenatal levels of androgen. Directly measuring prenatal androgen presents a challenge, but researchers use proxies. The most common being the ratio of the second to fourth digit (2D:4D)—there is evidence that it is associated with prenatal testosterone levels (Lutchmaya et al., 2004) and that the gender difference in this marker is of a medium effect size (Breedlove, 2017). Studies on the relationship between prenatal androgen exposure and homosexuality in men present an ambiguous picture (Swift-Gallant et al., 2023). A meta-analysis found no significant association between the digit ratio and non-heterosexuality in men (Grimbos et al., 2010). While some researchers concluded that this means no such relationship exists (Breedlove, 2017), others note that inconclusive results may be due to failure to use appropriate sub-groupings within the category of gay men or lack of investigation of potential interactions (Balthazart & Court, 2017; Skorska & Bogaert, 2017). Notably, there is evidence to suggest that both higher and lower-than-average levels of androgen activity during development can be associated with homosexuality in men, suggesting a possible curvilinear relationship (Bogaert & Hershberger, 1999; Skorska & Bogaert, 2017; Swift-Gallant et al., 2019a, b, 2021). Interestingly, gay men taking the top sexual role have been found to have male-typical digit ratios, while those taking the bottom role have a more feminized digit ratio—suggesting that only the latter group had been exposed to lower-than-average prenatal testosterone levels (e.g., Swift-Gallant et al., 2021). Associations have also been found between markers of prenatal androgen exposure and gender nonconformity (Auyeung et al., 2009). Interestingly, a meta-analysis revealed that sex differences are greater for the right than the left 2D:4D (Hönekopp & Watson, 2010). Another meta-analysis regarding the relationship between elevated androgen exposure due to congenital adrenal hyperplasia and the 2D:4D ratio in the case of men revealed a significant association only in the right hand (Richards et al., 2020), and a similar effect was suggested by a recent study directly measuring mothers’ testosterone in pregnancy and their son’s 2D:4D (Richards et al., 2022)*.* The above suggests that the right 2D:4D may be a better biomarker than the left 2D:4D.

### Fraternal Birth Order

The observation that gay men have more older brothers than heterosexual men has been robustly demonstrated (including a meta-analysis with a total sample of over 10,000 men (Blanchard, 2018). It has been proposed that this effect may be caused by a maternal immune response to Y-linked minor histocompatibility agents in a way similar to that observed in the hemolytic disease of the newborn (Blanchard & Ellis, 2001). The fetus’s blood passing through the placenta to the mother may stimulate an immune response to proteins coded on the Y chromosome, which is important for the fetus's sex differentiation in the male direction, stronger with every subsequent male pregnancy. An accumulation of antibodies against the Y-linked proteins in a pregnant mother’s organism may impact the function of proteins expressed in the brain during the process of sexual differentiation, causing partial feminization of the offspring, later manifested as sexual attraction toward men. Research suggests that the hypothesized immunization indeed takes place (James et al., 2003; Tan et al., 2008; Verdijk et al., 2004). Piper et al. (2007) reported that specific cytotoxic lymphocytes sensitive to a protein coded on the Y chromosome were found in about 37% of women who had had one male pregnancy and as many as 50% of women who had had at least two male pregnancies. Only one study analyzed this relationship directly—comparing immunization toward proteins coded on the Y chromosome, which are expressed in the brain of mothers of gay and heterosexual men, considering mothers’ total number of male pregnancies. Antibodies were found to be present in significantly larger quantities in mothers of gay men, with mothers of gay men who had the most older brothers being the most immunized (Bogaert et al., 2018). Research suggests that about 15–48% of gay men owe their sexual orientation to this effect (Blanchard & Bogaert, 2004; Cantor et al., 2002). These numbers might be underestimated (Bogaert & Skorska, 2011), as studies often do not take into account miscarriages, which are even more likely to lead to immunization (Bianchi et al., 2001). Moreover, for some women, one pregnancy is enough to elicit high immunization (Bogaert et al., 2018), suggesting a possible role of mothers' genetic makeup. In terms of associations between this marker and other markers, a study has found evidence for the fraternal birth order effect only among gay men who assumed a bottom anal sex role (Swift-Gallant et al., 2018), and an association with gender nonconformity (Swift-Gallant et al., 2019a, b).

### Familiality

Genetic mechanisms involved in sexual orientation can be investigated by measuring the aggregation of non-heterosexuality in families. A large body of studies on different populations demonstrated that non-heterosexuality clusters in families and male homosexuality are inherited through both paternal and maternal lines (Bailey et al., 1999, 2020; Camperio-Ciani et al., 2004; Hamer et al., 1993; Rahman et al., 2008; Schwartz et al., 2010; Semenyna et al., 2017; Vanderlaan et al., 2013a, 2013b). Twin studies also support the existence of genetic mechanisms (Alanko et al., 2010; Bailey et al., 2000; Kendler et al., 2000; Kirk et al., 2000; Långström et al., 2010; Santtila et al., 2008). In population-based twin studies, genetic factors were found to explain approximately 0.15–0.39 of the variance in sexual orientation in men (Alanko et al., 2013; Bailey et al., 2000; Långström et al., 2010). Interestingly, twins tend to also be similar in their levels of childhood gender nonconformity (Alanko et al., 2010; Dawood et al., 2000), and there is evidence that levels of heritability of childhood gender nonconformity may even be as high as 70% (Bailey et al., 2000; van Beijsterveldt et al., 2006). However, molecular studies remain scarce, and early studies provided rather inconclusive results, which could be related to the relatively small sample sizes (Ganna et al., 2019; Hamer et al., 1993; Hu et al., 1995; Mustanski et al., 2005; Rice et al., 1999; Sanders et al., 2015; Wang et al., 2012). Only recently, the emergence of enormous genetic databases such as the UK Biobank and 23 and Me allowed for performing analyses with a much higher power. Using these databases, the most extensive genome-wide association study on sexual orientation assessed that genetic factors account for 8 to 25% of the variation in sexual orientation, but their results also suggested that sexual orientation should not be considered on a single same-sex–opposite-sex continuum (Ganna et al., 2019). It is also possible that there exist indirect genetic factors, e.g., acting through maternal DNA, among them genes predisposing mothers to immunization to fetal antigens (Bocklandt et al., 2006; Dragan & Folkierska-Żukowska, 2023; Iemmola & Camperio Ciani, 2009). In any case, measuring the number of non-heterosexual individuals in one’s family remains the simplest and cheapest way to get insight into the possible heritable components in the development of human sexual orientation (Pillard & Bailey, 1998; Pillard & Weinrich, 1986).

### Childhood Gender Nonconformity

Childhood gender nonconformity (CGN) is a developmental factor linked to adult non-heterosexuality. The strong link between childhood gender nonconformity and adult non-heterosexuality has been robustly demonstrated by both retrospective (Rieger et al., 2008) and prospective (Li et al., 2017; Xu et al., 2020) studies. The association between gender nonconformity and homosexuality persists into adulthood (Li et al., 2017; Lippa, 2008; Rieger et al., 2008, 2010); however, the effect sizes are large regarding CGN (Bailey & Zucker, 1995) and moderate to large for adult gender nonconformity (Lippa, 2008; Rieger et al., 2010), suggesting that childhood gender nonconformity is a better marker than adult gender nonconformity (that may be more malleable to social pressures, Rieger et al., 2010). Importantly, there are data to suggest that childhood gender nonconformity in men may be associated with prenatal factors, such as lower prenatal androgen exposure (Auyeung et al., 2009; Shirazi et al., 2022; Swift-Gallant et al., 2022), and maternal immunity (Swift-Gallant et al., 2019a, b), as well as handedness (Kishida & Rahman, 2015; Swift-Gallant et al., 2017, 2019a, b), and sexual orientation familiality (Alanko et al., 2010; Bailey et al., 2020; Burri et al., 2015; Swift-Gallant et al., 2019a, b), warranting further investigation of the relationship between childhood gender nonconformity and other biomarkers of sexual orientation.

As can be seen in the literature reviewed above, many studies have considered more than one of the factors at a time. However, studies that comprehensively analyze all of those factors are still scarce. The authors know of one study to date that evaluated a number of such biodevelopmental markers within the same individuals—heterosexual and non-heterosexual men (Swift-Gallant et al., 2019a, b). This study considered three markers, namely the proportion of older brothers, handedness, and familiality. Using a latent profile analysis, six profiles of men were defined: a group without biomarkers, a group characterized by a higher proportion of older brothers, a group associated with non-right-handedness, a group characterized by high levels of childhood gender nonconformity, a group characterized by the most masculine digit ratio, and a group where non-heterosexuality was prevalent in the family. The groups were then compared in terms of sexual orientation and gender nonconformity. While most men independent of sexual orientation were in the first profile (that was also the most gender-conforming), the profiles associated with biomarkers were composed largely of non-heterosexual men.

In the current study, we collected an even larger set of biodevelopmental markers: the ratio of the 2nd and 4th digits of both hands (2D:4D ratio), the proportion of older brothers, gender nonconformity in childhood, handedness, and familiality. Because of its interactions and associations with various other biomarkers of non-heterosexuality in men, in contrast to the previous LPA study (Swift-Gallant et al., 2019a, b), we decided to treat childhood gender nonconformity as a biodevelopmental marker and include it in the LPA analysis. The aims of this study were to (1) investigate whether gay and straight men differ across established markers of male sexual orientation: digit ratio, the proportion of older brothers, childhood gender nonconformity, and handedness; (2) identify latent profiles based on those markers; and (3) identify whether the proportions of gay and straight men differ within the classes.

## Method

### Participants

A total of 957 cisgender men aged 18–63 (*M* = 28.05 years, *SD* = 6.9) took part in the study. They were either gay (*n* = 519) or straight (*n* = 438). Data were collected between December 2016 and January 2020.

Participants were recruited in Poland via an online survey completed as a part of a larger project concerning correlates of sexual orientation in men. The survey was distributed through Facebook (paid adverts and organic traffic), leaflets, posters, and word of mouth. This was completed by 3515 individuals between December 2016 and January 2020. Of these, 2285 fulfilled the inclusion criteria (being a predominantly heterosexual or predominantly homosexual cisgendered man and aged 18–45 years) and were invited to the laboratory. The final sample consisted of 957 participants (519 gay and 438 heterosexual men). All participants who attended the laboratory received remuneration (approximately 10 USD).

### Measures

#### Sex Assigned at Birth and Gender Identity

Sex assigned at birth and gender identity were measured using the “two-step” approach (Conron et al., 2014) with questions about sex assigned at birth and current gender identity, i.e., “What sex were you assigned at birth?” (possible answers: “male,” “female”) and “What gender do you currently identify with” (possible answers: “man,” “woman,” “transgender,” “I do not identify as either woman or man or transgender”). Only cisgender men (i.e., men who reported being assigned male at birth and currently identified as a man) took part.

#### Sexual Orientation

Sexual orientation was assessed using a Polish adaptation of the Sell Assessment of Sexual Orientation (Folkierska-Żukowska et al., 2020; Sell, 1996), measuring three dimensions of sexual orientation: sexual attractions, sexual contact, and sexual identity, separately for two target genders (men and women). Here, we defined predominantly heterosexual men as those who had been sexually attracted to 0 or 1 man in the past year, who had never been attracted to a man or were attracted to a man less than once per month, whose attraction to a man in the past year ranged from “not at all” to “mildly”; who had been sexually attracted to at least one woman in the past year; who had been sexually attracted to a woman at least once a month; and who identified as “not at all homosexual” or “slightly homosexual” and as either “very heterosexual” or “extremely heterosexual.” Criteria for predominantly homosexual men were, *mutatis mutandis*, identical. Questions about sexual contact were not included in our criteria due to the limitation of being potentially constrained by social censure of same-sex sexuality.

#### Recalled Childhood Gender Nonconformity

Recalled childhood gender nonconformity was measured using a Polish adaptation of the Recalled Childhood Gender Identity/Gender Role Questionnaire (Folkierska-Żukowska et al., 2020; Zucker et al., 2006). Only items corresponding to Factor 1 of the scale (concerning gender conformity) were analyzed. An example of an item (and its associated point score) would be “As a child, the characters on TV or in the movies that I imitated or admired were: a. always girls or women (1), b. usually girls or women (2), c. girls/women and boys/men equally (3), d. usually boys or men (4), e. always boys or men (5), f. I did not imitate or admire characters on TV or in the movies.” A mean score was calculated for each participant for which the absolute range was 1–5, with 1 corresponding to extremely feminine behavior and 5 to extremely masculine behavior. A response of “I did not do this/feel this way” to an item was omitted. Since the questioning of traditional gender roles has intensified in recent years, for clarity, we modified the instructions on the questionnaire by adding “In the case of the words ‘feminine’ and ‘masculine,’ we refer to their stereotypical definitions functioning in society.”

#### Handedness

Laterality index (handedness) was measured using a Polish adaptation of the Fazio Laterality Inventory (Dragan et al., 2022; Fazio et al., 2013). On this scale, participants answer about the percentage of time (0 to 100%) that they use their right hand to perform the following common actions: writing, drawing, waving hello or goodbye, using a TV remote, snapping fingers, scratching an itchy nose, pointing at something in the distance, throwing an object, reaching to pick up an object, and using a hammer.

#### Proportion of Older Brothers

The proportion of older brothers was calculated and corrected for family size using the formula proposed by Blanchard (Blanchard, 2014): (*n older brothers* + 0.25)/(*n siblings* + 1), based on questions regarding the number of younger and older brothers and sisters.

#### Familiality

Familiality scores were calculated based on an open-ended question regarding non-heterosexual individuals in participants’ families. A participant would get a score of 1 for each reported male non-heterosexual member of the family and 0.5 for each female non-heterosexual member of the family (e.g., the score would be 1.5 if they reported a male and a female non-heterosexual in their family).

#### 2D:4D Digit Ratio

Scans of participants’ left and right hands were collected in the lab. Participants were excluded case by case from the measurement in the event of reporting previous fractures, exhibiting deformities, missing one of the hands etc. For each scan, three measurements of 2nd and 4th digit were performed in ImageJ software (Schneider et al., 2012), the average of which was used to calculate the 2D:4D digit ratio (Robinson & Manning, 2000).

All questionnaires were filled out online. Participants received remuneration of 36 PLN (approximately 7 USD) for taking part in the meeting, where hand scans were collected.

### Procedure

We successfully collected data on handedness and childhood gender nonconformity from 100% of the participants. A total of 764 (80%) participants answered the questions needed to calculate the proportion of older brothers. Hand scans for measurements to calculate the digit ratio were collected for the left hand from 689 (72%) and for the right hand from 692 (72%) participants. Two left-hand and six right-hand scans were excluded due to the piece of paper with the participant’s number and date of scan obstructing at least one finger, making the measurement impossible. To ensure the highest possible quality of data, further 86 left-hand and 68 right-hand scans were excluded due to participants’ fingers not lying fully flat on the scanner. Thus, the final dataset consisted of measurements of the left hand from 620 (65%) participants and the right hand from 600 (63%) participants.

With regard to the items used to calculate the familiality score, 758 (80%) of participants answered the question. Among the participants who answered, 72 (8%) participants answered that they “don’t know,” 457 (49%) participants gave a clear “no,” and another 116 (12%) answered with a tentative “no” (e.g., “probably not,” “no one has admitted to it,” “not that I know of,” “I think not”; “I don’t think so, but I can’t exclude that someone hides their non-heterosexuality”), 8 (~ 1%) answers were just participants’ suspicions (e.g., “my father may be gay,” “I suspect that my cousin may be gay”). All those answers received a score of 0. 105 (11%) participants identified non-heterosexual family members.

Importantly, Little’s test failed to reject the null hypothesis that the data was missing completely at random, *χ*^*2*^ (28) = 55.76, df = 28, *p* = 0.061, which is a prerequisite for LPA analysis with missing data as well as missing variable imputation (Little, 1988; Little & Rubin, 2019; Pedersen et al., 2017).

### Statistical Analyses

The distribution of each variable was tested using the Kolmogorov–Smirnov test, and group comparisons on the variables of interest were performed in SPSS ver. 29, using Student’s *t*-test or Kruskal–Wallis H test, depending on whether the distribution was normal or not.

To group the sample based on the collected measures, latent profile analysis was conducted in R software using the tidyLPA package (Rosenberg et al., 2018), which is based on the mclust package (Scrucca et al., 2023). To deal with the missing data, we first verified that the data were missing completely at random (MCAR) using Little’s MCAR test (Little, 1988) in SPPS ver 29. Then we used the “imputeData” function of the mclust package in R for missing data imputation. An analytic hierarchy process based on the fit indices: Akaike information criterion (AIC), approximate weight of evidence (AWE), Bayesian information criterion (BIC), classification likelihood criterion (CLC), and Kullback information criterion (KIC; Akogul & Erisoglu, 2017) was used to choose the best solution model.

Chi-square test and post hoc 2-tailed pairwise Z-tests with Bonferroni correction were performed in SPSS ver. 29 to analyze the difference in the proportion of gay and straight men in each of the identified classes.

## Results

**Fig. 1:**
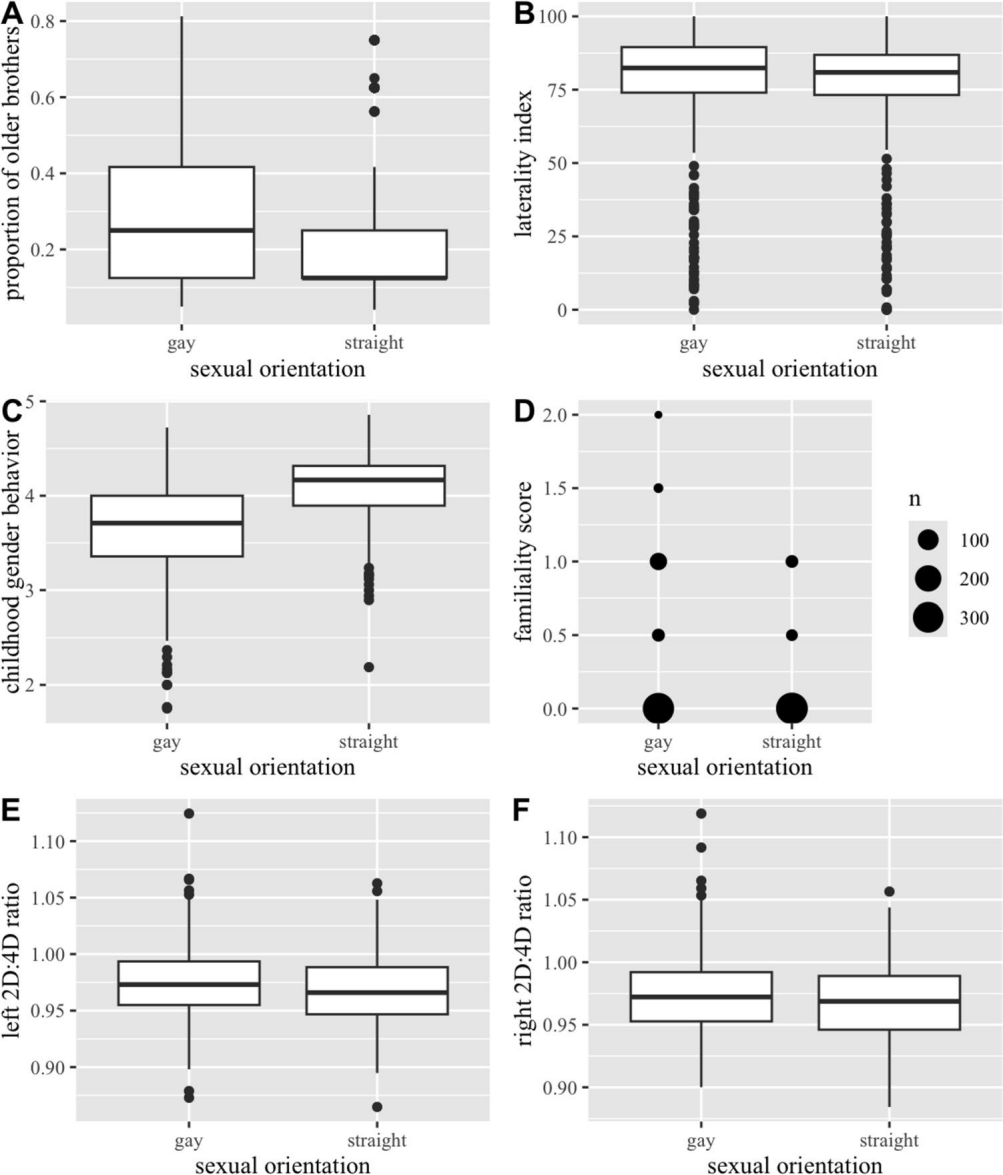
Distribution of the values for each of the biomarkers, for each group. Proportion of older brothers, laterality index, childhood gender behaviors, and digit ratios **A**–**C**, **E**–**F** are presented using boxplots. Distribution of the familiality scores, **D** is represented using a count plot due to the extremely skewed distribution of this variable, which makes a box plot less informative

Gay men were significantly more gender nonconforming in childhood than heterosexual men, *t*(911.07) = 16.28, *p* < 0.001. They also had significantly more older brothers, *t*(756.37) = 3.02, *p* < 0.01, were less likely to be left-handed, *H*(1) = 6.50, *p* < 0.05, and had more non-heterosexual family members, *H*(1) = 23.84,* p* < 0.001 — only 8% of straight men reported non-heterosexual family members in contrast to 19% of gay men. In the case of digit ratios, we found a significant difference between heterosexual and gay men only in both the right, *t*(598) = 2.70, *p* = 0.004, and the left hand, *t*(618) = 2.90, *p* = 0.002. Graphical representation of the distribution of values for each of the variables in the two groups can be found in Fig. 1. In line with the findings suggesting that right 2D:4D is a more robust biomarker (Hönekopp & Watson, 2010; Manning et al., 1998; Richards et al., 2020, 2022), we only included the data for the right hand in our further analyses.

The next step was to perform the LPA analysis, including the following markers: handedness, 2D:4D ratio, familiality, proportion of older brothers, and childhood gender nonconformity. There are several approaches to choosing a model in LPA analysis, including statistical tests of model fit, choosing a model with no small classes (e.g., less than 5% of the sample), and choosing a model that is simply best suited to the researchers’ needs (Nasserinejad et al., 2017). In the case of our analysis, we used an analytic hierarchy process built into the tidyLPA package in R, based on the fit indices (Akogul & Erisoglu, 2017). These indices suggested that the model with 6 profiles was the best solution (Table 1).

**Table 1:** Comparison of the fit of latent profile analysis models with 1–7 profiles

	AIC	AWE	BIC	CLC	KIC	*p*-value (BLRT)
1-profile	13,594.23	13,739.51	13,642.87	13,576.23	13,607.23	
2-profile	12,842.53	13,076.24	12,920.35	12,812.47	12,861.53	0.01
3-profile	12,605.75	12,927.94	12,712.75	12,563.57	12,630.75	0.01
4-profile	12,610.27	13,021.34	12,746.45	12,555.56	12,641.27	0.22
5-profile	12,107.67	12,606.99	12,273.04	12,041.08	12,144.67	0.01
6-profile	12,069.04	12,656.73	12,263.59	11,990.45	12,112.04	0.01
7-profile	12,081.43	12,757.70	12,305.17	11,990.64	12,130.43	0.98

AIC: Akaike information criterion; AWE: Approximate weight of evidence; BIC: Bayesian information criterion; CLC: Classification Likelihood Criterion; KIC: Kullback information criterion. BLRT: bootstrapped likelihood test

For each of the approximate fit indices (AIC, AWE, BIC, CLC, KIC) a lower value indicates a better fit

**Fig. 2:**
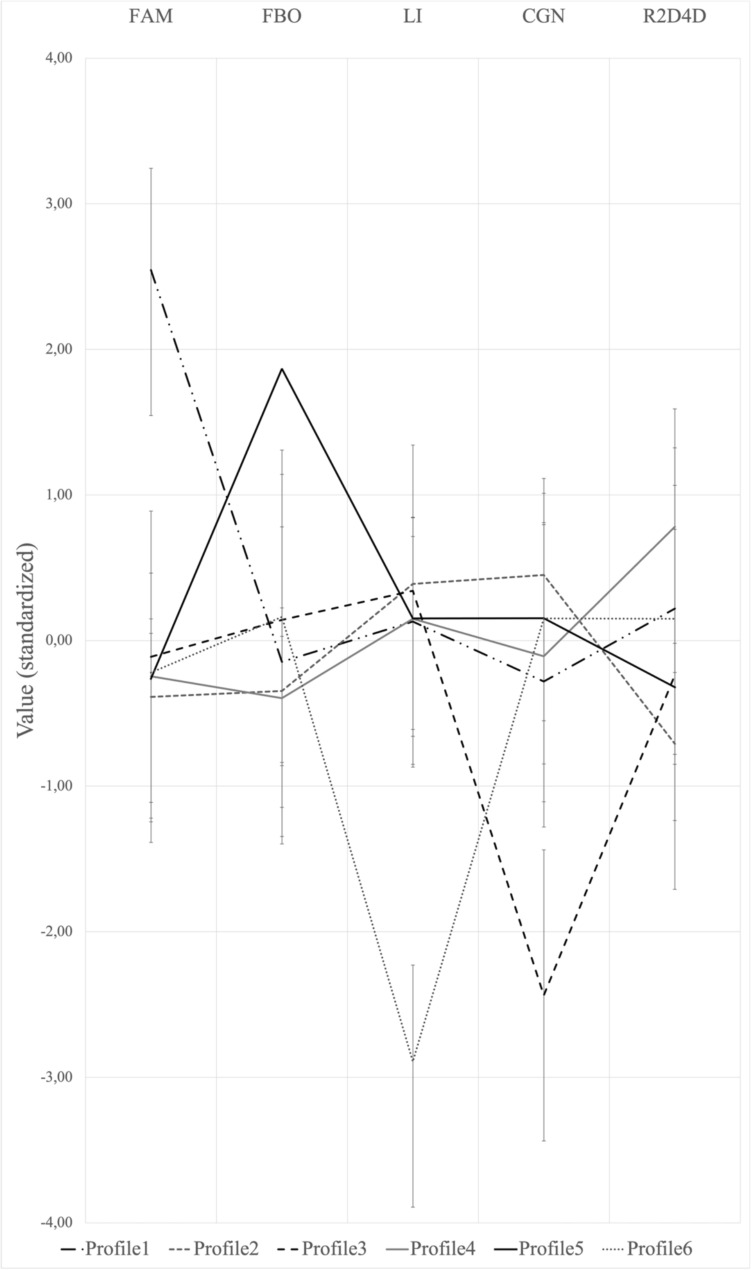
Profiles identified by the LPA analysis based on the five markers. Y-axis represents distance from the mean in standard deviations (0–standardized mean). R2D:4D–digit ratio in the right hand, FBO–proportion of older brothers, CGN–childhood gender nonconformity (higher scores correspond to higher masculinity and lower scores correspond to higher femininity, i.e., lower scores indicate higher nonconformity), LI–laterality index (handedness, the higher the score the more right-handed the individual)

Following that, we verified the proportions of gay and straight men that fell into each of the profiles. The first profile, associated with the familiality index, counted 102 individuals and significantly more gay than straight men (15% vs. 5%). The second profile contained 313 participants who had the most masculine digit ratios, the most masculine behavior in childhood, and were the most right-handed. This group was composed of a higher proportion of straight than gay men (42% of all heterosexual men and 25%, respectively, of homosexual men fell into this profile). The third profile, characterized by most childhood gender nonconformity, contained 45 individuals, most of whom were gay (8% of gay men vs. 1% of straight men). The fourth profile, characterized by the most feminized digit ratio, contained 306 individuals, and there was no significant difference in the proportion of gay and straight men (32% and 33%, respectively). The fifth profile, associated with a high proportion of older brothers, contained 121 participants, and there was no significant difference in the proportion of gay and straight men (13% vs. 12%, respectively). The sixth profile was composed of 74 left-handed individuals, and there was no significant difference in the proportion of gay and straight men (both 8%; Table 2, Fig. 2).

**Table 2:** Proportion of gay and straight men falling into each class, and their comparison using pairwise Z-tests

	Heterosexual, n = 438		Gay, n = 519		Total		*p*-value
	N	%	N	%	N	%	
Profile 1	22	5.02	76	14.64	98	10.24	< .05
Profile 2	182	41.55	131	25.24	313	32.71	< .05
Profile 3	4	0.91	41	7.90	45	4.70	< .05
Profile 4	144	32.88	162	31.21	306	31.97	n.s
Profile 5	51	11.64	70	13.49	121	12.64	n.s
Profile 6	35	7.99	39	7.51	74	7.73	n.s

We also performed post hoc analyses of CGN levels across the groups (using the z-scores). One-way ANOVA revealed a significant difference between the groups, *F*(5, 951) = 108.12, *p* < 0.001; Tukey’s HSD test has revealed that profile 3 had the lowest value (-2.43), followed by profile 1 (-0.28), 4 (-0.10), 6 (0.15), 5 (0.15), and 2 (0.44). See Table 3 for the summary of differences between the groups.

**Table 3:** Tukey HSD post hoc analysis of differences in CGN levels across the profiles identified by CGN. Each column represents homogenous subsets. Z-scored means are displayed for each profile

	Subset for alpha = 0.05				
	N	1	2	3	4
Profile 3	45	− 2.43			
Profile 1	98		− 0.28		
Profile 4	306		− 0.10	− 0.10	
Profile 6	74			0.15	0.15
Profile 5	121			0.15	0.15
Profile 2	313				0.44
*p-value*		1.00	0.65	0.19	0.09

## Discussion

In this study, we collected several biomarkers that have been previously shown to be associated with male non-heterosexuality. Low gender conformity in childhood is a robust developmental correlate of adult non-heterosexuality (Rieger et al., 2008), higher 2D:4D ratio a biomarker of lower prenatal testosterone exposure (Lutchmaya et al., 2004), higher proportion of older brothers is a biomarker of maternal immunization to Y-linked antigens (causing undermasculinization of the brain; Bogaert et al., 2018), and non-right-handedness and extreme right-handedness are associated with non-heterosexuality (Bogaert et al., 2007; Ellis et al., 2017; Kishida & Rahman, 2015; Lalumière et al., 2000), with non-right-handedness being additionally associated with gender nonconformity (Swift-Gallant et al., 2017). Non-heterosexuality has also been previously shown to aggregate in families (e.g., Bailey et al., 1999), suggesting a genetic component. The aims were to (1) see whether gay and heterosexual (straight) men differ on those markers, (2) identify latent profiles based on those markers, and (3) see whether the proportions of gay and straight men differ within the profiles.

With regard to the first aim, gay men reported being less gender-conforming in childhood, had a higher proportion of older brothers, were more right-handed, had more non-heterosexual relatives, and had more feminized digit ratios on both their left and right hand (Fig. 1). However, in line with the findings suggesting that right 2D:4D is a more robust biomarker (Hönekopp & Watson, 2010; Manning et al., 1998; Richards et al., 2020, 2022), we only included the data for the right hand in our further analyses.

As the next step we explored whether immunological mechanisms, genetic mechanisms and hormonal mechanisms constitute diverging pathways to male non-heterosexuality. Using latent profile analysis (LPA) we identified six profiles based on the collected variables (Fig. 2). The most numerous profile (profile 2, *n* = 313) was associated with the most masculine digit ratios, the most masculine behavior in childhood, and the most right-handed. A significantly higher proportion of straight men than gay men were classified in this profile. About 42% of straight men and only about 25% of gay men belonged to that profile. The second most numerous profile (profile 4, *n* = 306) consisted of participants who had the most feminine digit ratio and scored around average on all other measures. This profile was also significantly more gender nonconforming in childhood than profile 2 but less gender nonconforming than profile 1. Surprisingly, this profile did not differ in terms of the proportion of gay and straight men in it. The profile associated with the highest proportion of older brothers (profile 5,* n* = 121) was the third most numerous and also did not differ in terms of the proportion of gay and straight men in it. It was relatively gender conforming, with masculinity levels no different from profile 2, 4 and 6, but significantly higher than in profiles 1 and 3. Following that was the profile associated with familiality (profile 1, *n* = 98), which contained significantly more gay men (15% vs. 5%). Interestingly, this profile was also the second most gender nonconforming in childhood (right after profile 3, and no different to profile 4, but significantly more gender nonconforming than all other profiles). This is in line with the reports indicating shared genetic factors for childhood gender nonconformity and sexual orientation (Alanko et al., 2010). The profile associated with left-handedness (profile 6, *n* = 74) and average levels of all other biomarkers did not differ in terms of the proportion of gay and straight men in it. It was one of the more childhood gender-conforming profiles. On the other hand, the smallest profile (profile 3, *n* = 45), associated with the most feminine childhood gender behaviors (significantly different from all other groups) consisted of almost only gay men—only 1% of straight men fell into that group, in contrast to 8% of gay men, making in the most gay-dominated profile.

The fact that no significant differences in the proportions of gay and straight men were found in the group with the highest proportion of older brothers is surprising, especially since initial group comparisons suggested that gay men were more likely to have a higher proportion of older brothers (Fig. 1). It is possible that a higher proportion of older brothers coincided with other biomarkers in some of the gay men, and these men were classified in other profiles. It has been previously found that this biomarker is associated with higher femininity (Swift-Gallant et al., 2019a, b; Swift-Gallant et al., 2019a, b). While there is no strong evidence for such association in our sample, it should be noted that individuals in the profile associated with the most childhood femininity scored slightly above average on FBO. Another group that scored above average is the one consisting of left-handed individuals, which contrasts with previous findings suggesting that individuals with older brothers are less likely to be left-handed (Blanchard et al., 2006).

Contrary to some reports (e.g., Lalumière et al., 2000), we found no evidence that left-handedness is more prevalent in gay men. However, notably, the most extreme right-handedness was present in the group who also had the most masculine digit ratios (suggesting the highest prenatal testosterone) and was the most masculine in childhood. Interestingly, while the profile with the most masculine 2D:4D ratio was also the most gender-conforming and predominantly heterosexual, the profile that was the most gender nonconforming in childhood also had one of the most masculine digit ratio scores. This seems in line with there being a curvilinear rather than linear relationship between prenatal testosterone levels and childhood gender behaviors.

Interestingly, only the familiality measure was associated with elevated levels of gender nonconformity. However, it should be noted that the gender nonconformity measure used in this study offers a combined score relating to both feminine and masculine behaviors, which may result in a loss of information. In the previous LPA study, the profile associated with immunological factors was composed of participants who had typical levels of masculinity, but were also the most feminine, suggesting that considering masculinity and femininity separately could yield different results (see: Swift-Gallant et al., 2019a, b).

Individuals who were gender nonconforming in childhood constituted an entirely separate profile, falling around average on all other measures, and with digit ratios that were not indicative of lower prenatal androgen exposure. This is a surprising result considering the previously reported associations between childhood gender nonconformity and most other biomarkers—including feminized digit ratios (e.g., Auyeung et al., 2009), handedness (e.g., (Kishida & Rahman, 2015; Swift-Gallant et al., 2017, 2019a, b), maternal immunity (Swift-Gallant et al., 2019a, b), and familiality (Alanko et al., 2010).

It is worth remarking that while the majority of gay men were spread across groups with all possible biomarkers of feminization or genetic influences, the majority of straight men belonged to the one group characterized by levels of the biomarkers that would be associated with the highest masculinization (and no genetic influences), and only quarter of gay men belonged to that profile. When considering this subgroup of gay men, it needs to be noted that the biomarkers we used are not perfect and do not allow us to fully capture the presence or lack thereof of the underlying phenomena. For instance, immunization has also been observed in mothers of gay sons with no older brothers (Bogaert et al., 2018), and thus, immunization could still be at play in some of the gay men in that group of gay men. Notably, the prevalence of non-heterosexuality in our participants’ families could have been underestimated (an issue further explored in Limitations). And so, this group could also contain participants unaware of having non-heterosexual relatives. Moreover, the current study did not exhaust all possible biomarkers. Epigenetic (Ngun & Vilain, 2014), alternative immunological (Blanchard, 2012), as well as yet unknown factors, could be at play in this group of gay men.

Conversely, it should also be noted that over half of heterosexual men in our sample did show biomarkers that the literature associates with non-heterosexuality. And while there were almost no straight men with high levels of gender nonconformity or with non-heterosexual family members, there was no difference in the proportion of gay and straight men who fell into the groups characterized by the high proportion of older brothers and by feminized digit ratios. This indicates that the mechanisms behind these biomarkers may not be sufficient for the development of non-heterosexual sexual orientation. Again, it could be that some of the hereby uncaptured or unknown mechanisms could play a role in tipping over the scale in the direction of non-heterosexuality. It is also possible that there could be a better model to describe our data to see the interplay between the collected biomarkers. When performing an LPA analysis, one runs a risk of overfitting the model. The hereby presented model with six groups was selected using multiple statistical fit indices together in a hierarchical process. This method favored profiles where only one of the biomarkers had either particularly elevated or decreased levels. However, there are other possible methods of model selection, and choosing the best one remains challenging. We cannot exclude that if a model with a lower number of classes was selected, we would see more convergence between the biomarkers and thus gain more insight into the interplay between them.

### Limitations

The study is not without certain limitations. Our measure of childhood gender nonconformity was retrospective, which can introduce recall and other biases. However, it is noteworthy that retrospective assessments of childhood gender nonconformity are reported to be highly reliable (Bailey & Zucker, 1995; Li et al., 2017; MacMullin et al., 2021; Rieger et al., 2008). Another limitation of this measure is that it produces a score that considers feminine and masculine behaviors together, while there are indications to suggest that considering them separately may be more informative (Martin et al., 2017; Swift-Gallant et al., 2019a, b). Similarly, in the way we calculated sexual orientation, it was treated as a single dimension, while there exists evidence that androphilia and gynephilia are only moderately correlated in males and very weakly correlated in females (Lippa & Arad, 1997; Shirazi et al., 2021; Swift-Gallant et al., 2023). The use of the 2D:4D ratio as a proxy for prenatal androgen exposure is controversial (Hampson & Sankar, 2012). However, we believe that the long history of its use in studies regarding sexual orientation is enough of a reason to include it in analyses like those presented here. It is also worth mentioning that this measure may depend not only on androgen exposure but also on androgen sensitivity, which would not be captured by measuring levels of prenatal testosterone (Knickmeyer et al., 2011; Manning et al., 2003). Another limitation of this measure is that it does not capture the whole hormonal milieu, and fails to consider a possible role of estrogens in androphilia (Shirazi et al., 2021; Swift-Gallant et al., 2023). There were some challenges regarding collecting data to calculate familiality—a high number of answers were tentative or indicated a lack of such knowledge, which may indicate a potential underestimation of the prevalence of non-heterosexuality in our participants’ families. However, this challenge was not due to the questions in the survey having been poorly designed, but rather a reflection of the fact that non-heterosexuality is still a taboo in Poland, a country repeatedly reported as the least LGBTQIA+ friendly in the European Union (Ilga-Europe, Annual Report 2024, 2024), and many people, especially in the older generations hide their sexual orientation. Three of our measures (proportion of older brothers, familiality and 2D:4D) contained missing data. However, LPA is a method that is well-equipped to deal with missing data, and it is a common practice to use it to analyze similar datasets. It should be noted that LPA analysis assumes that the data is missing at random (Ferguson et al., 2020). We performed statistical tests that suggest the data might not be missing completely by random—while the *p*-value of 0.061 fulfills the conventional cut-off point of 0.05, this may suggest some probabilistic relationship between missing data and other variables (see, e.g., Kyriacou, 2016 for limitations of rigid *p*-value cutoffs). However, we used imputation in R to deal with the missing data which deals with this limitation.

### Conclusion

The presented results are in line with previous findings, suggesting that there may exist multiple distinct biodevelopmental pathways that influence same-sex sexual orientation in men and that factors influencing sexual orientation may interact or converge. In particular, our results suggest that the immunological, genetic, and hormonal mechanisms seem to be at least partially independent. Notably, the majority of gay men fell into profiles with high levels of biomarkers associated with feminization and non-heterosexuality running in the family, and the majority of straight men belonged to the one group characterised by biomarkers of masculinization (and no genetic influences), while only quarter of gay men belonged to that profile. It needs to be noted that factors that were not measured here could be at play, or there may be undetected or unknown factors that are also involved. Further investigations of biological differences within sexual orientation categories are warranted to untangle the complex interplay between the genetic, epigenetic, prenatal, and postnatal (including social) factors.
